# Differences in Hebbian stimulation effects between biceps and triceps brachii in humans

**DOI:** 10.1152/jn.00168.2025

**Published:** 2025-07-21

**Authors:** Carley L. P. Butler, Minkyu Lee, Monica A. Perez

**Affiliations:** 1 Shirley Ryan AbilityLab, Chicago, Illinois, United States; 2 Department of Biomedical Engineering, Northwestern University, Evanston, Illinois, United States; 3 Department of Physical Medicine and Rehabilitation, Northwestern University, Chicago, Illinois, United States; 4 Edward Hines Jr. VA Hospital, Hines, Illinois, United States

**Keywords:** corticospinal pathway, motoneurons, neural plasticity, spinal synapses, upper limb muscles

## Abstract

Animal and human studies indicate that monosynaptic corticospinal connections are more prevalent in biceps than triceps brachii motoneurons. Based on this evidence, we hypothesized that Hebbian stimulation, which targets corticospinal-motoneuronal connections, would enhance corticospinal excitability more in the biceps than the triceps brachii. To test this hypothesis, we assessed motor-evoked potential (MEP) size using transcranial magnetic stimulation (TMS) at resting motor threshold (MEP-RMT) and maximum stimulator output (MEP-100%) immediately and up to 30 min poststimulation. During Hebbian stimulation, 180 paired pulses were delivered, with corticospinal volleys evoked by TMS arriving at corticospinal-motoneuronal synapses 1–2 ms before antidromic potentials from brachial plexus electrical stimulation. Central and peripheral conduction times were similar between muscles. We found that both MEP-RMT and MEP-100% increase in the biceps and triceps immediately and up to 30 min poststimulation. The increase in MEP-RMT was greater in the biceps compared with the triceps, whereas MEP-100% changes did not differ between muscles. Since the maximum MEP size was larger in the biceps than in the triceps, we conducted a control experiment testing responses at an intermediate size between MEP-RMT and MEP-100% (MEP-Control), ensuring similar baseline sizes between muscles. Notably, Hebbian stimulation continued to produce a greater increase in MEP-Control in the biceps than in the triceps. These findings suggest that Hebbian plasticity enhances corticospinal excitability more in the elbow flexor than extensor muscles, emphasizing the need to consider muscle-specific innervation patterns when future studies assess the therapeutic effect of this technique in individuals with motor impairment.

## NEW & NOTEWORTHY

Differences in corticospinal projections to elbow flexor and extensor motoneurons are well established. Our findings demonstrate that Hebbian stimulation, based on spike-timing-dependent plasticity, enhances corticospinal excitability more in the biceps brachii than in the triceps brachii. These results highlight the importance of incorporating muscle-specific innervation patterns when designing plasticity-driven therapeutic strategies.

## INTRODUCTION

Anatomical and electrophysiological studies have revealed differences in the neural control of elbow flexor and extensor muscles, despite the largely overlapping locations of corticospinal projections (1) and motoneurons (2) that innervate these muscles. For example, studies utilizing peristimulus time histograms (PSTHs) have demonstrated that monosynaptic corticospinal facilitation is more pronounced in elbow flexor motoneurons than in elbow extensor motoneurons (3). In addition, motor-evoked potentials (MEPs) elicited by transcranial magnetic stimulation (TMS) are typically larger in the biceps brachii than in the triceps brachii (4, 5). Furthermore, previous results support the presence of strong cortical inhibitory input to corticospinal projections controlling elbow extensor compared to elbow flexors muscles (6). However, the extent to which corticospinal projections to biceps and triceps brachii motoneurons are sensitive to plasticity remains largely unknown.

Hebbian stimulation is a protocol that can be used to examine plasticity in corticospinal projections to biceps and triceps brachii motoneurons. This technique has emerged as a promising strategy for enhancing sensorimotor function and improving the quality of life in individuals with upper motor neuron lesions (7–9). Based on the principles of spike-timing-dependent plasticity (STDP), Hebbian stimulation follows the concept that “neurons that fire together, wire together” (10), wherein a presynaptic terminal is repeatedly activated a few milliseconds before or after a postsynaptic terminal (11, 12). Differences in the efficacy of Hebbian stimulation in modulating corticospinal excitability have been reported across muscle groups. For example, a greater number of paired pulses is required to induce changes in corticospinal excitability in lower limb muscles compared with upper limb muscles (12, 13). Studies pairing auditory and muscle stimulation targeting brainstem pathways, by using STDP principles, have shown increases in corticospinal excitability in elbow flexor muscles but not in elbow extensor muscles (14, 15). Moreover, when plasticity is induced by sensory input, pairing motor point stimulation of a finger flexor or extensor muscle with TMS of the motor cortex enhances corticospinal excitability more in flexor muscles than in extensor muscles (16). Overall, repetitive practice of a tracking task involving finger flexor and extensor muscles resulted in increases in corticospinal excitability in flexor muscles but not in extensor muscles (17). It remains unclear whether similar differences are also present between more proximal flexor and extensor muscles. Based on this evidence, we hypothesized that Hebbian stimulation, which targets corticospinal-motoneuronal connections, would enhance corticospinal excitability more in the biceps than in the triceps brachii.

To test this hypothesis, we compared changes in corticospinal excitability between the biceps brachii and triceps brachii using TMS at resting motor threshold (MEP-RMT) and maxi-mum stimulator output (MEP-100%) immediately and up to 30 min after Hebbian stimulation targeting corticospinal-motoneuronal synapses. Hebbian stimulation consisted of 180 paired pulses, with corticospinal volleys evoked by TMS arriving at corticospinal-motoneuronal synapses 1–2 ms before antidromic potentials from brachial plexus electrical stimulation.

## MATERIALS AND METHODS

### Subjects

Fourteen neurologically intact individuals (mean age 27.1 ± 4.5 yr, 8 males and 6 females) participated in the study. All participants gave written informed consent to the experimental procedures, which were approved by the local ethics committee at Northwestern University and performed following the Declaration of Helsinki. Ethical approval for this study was obtained from the Institutional Review Board (STU00210458).

### Electromyographic Recordings

Electromyogram (EMG) was recorded from the biceps and triceps brachii of the dominant right side through bipolar surface electrodes (Ag-AgCl, 10 mm diameter, 1 cm apart) secured on the skin over the belly of the muscle. The signals were amplified, filtered (30–2,000 Hz), and sampled at 4 kHz for offline analysis (CED 1401 with Signal software, Cambridge Electronic Design, Cambridge, UK).

### Experimental Setup

**Figure 1.**
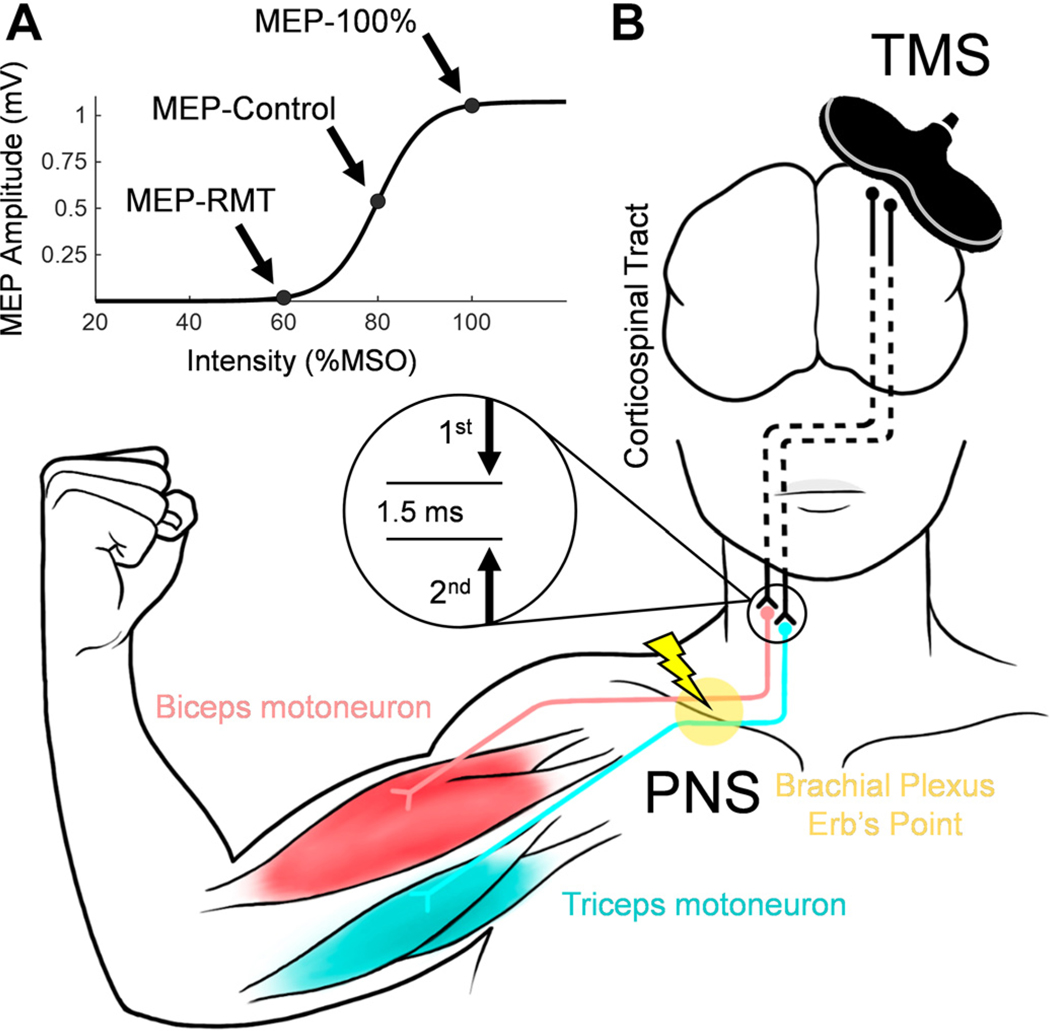
Experimental setup. *A*: baseline motor-evoked potential (MEP) size measurements were assessed at various points along the input-output excitability curve. The *x*-axis represents stimulus intensity as a percentage of maximum stimulator output (%MSO), whereas the *y*-axis represents response amplitude in millivolts (mV). Example MEP baseline target sizes, indicated by black arrows, included the resting motor thresh-old (MEP-RMT), maximum intensity (MEP-100%), and an approximate mid-point (MEP-Control). *B*: illustration of the Hebbian stimulation protocol. Suprathreshold transcranial magnetic stimulation (TMS) was applied over the arm representation of the primary motor cortex to activate corticospinal neurons (black lines) projecting to biceps and triceps brachii moto-neurons (biceps motoneurons in pink and triceps motoneurons in blue). Suprathreshold peripheral nerve stimulation (PNS), represented by a lightning bolt symbol, was delivered via supramaximal stimulation to the Erb’s point at the brachial plexus (yellow circle). The circular callout depicts the interstimulus interval (ISI) between paired pulses, ensuring that descending volleys elicited by TMS (first black arrow) reached the presynaptic terminal of corticospinal neurons 1–2 ms before antidromic PNS volleys arrived at the postsynaptic terminal in spinal motoneurons (second black arrow).

Subjects were seated in an armchair with their arm supported by a custom platform, positioning the shoulder and elbow at 90°, and the tested arm was secured with straps to ensure stable isometric contractions. During maximum voluntary contraction (MVC) measurements, subjects were instructed to perform isometric elbow flexion for the biceps brachii and isometric elbow extension for the triceps brachii while force and EMG signal were recorded. Three MVC measurements were performed for each muscle, each lasting 3–5 s, with at least 1 min of rest between measurements. The maximal mean EMG activity from the rectified response for over 1 s for each MVC trial was deter-mined, and the highest value from the three trials was used for each muscle. We found that average EMG (biceps = 0.74 ± 0.46 mV, triceps = 0.50 ± 0.37 mV; *P* = 0.14) and force (biceps = 216.6 ± 74.5 N, triceps = 195.3 ± 64.5 N; *P* = 0.43) MVC values were not significantly different between the biceps and triceps. Similar results were observed when the analysis was based on the largest MVC values measured using EMG (biceps = 0.77 ± 0.53 mV, triceps = 0.46 ± 0.34 mV; *P* = 0.08) or force (biceps = 212.3 ± 75.8 N, triceps = 196.0 ± 64.4 N; *P* = 0.44). During Hebbian stimulation, TMS and peripheral nerve stimulation (PNS) were delivered to target corticospinal-motoneuronal connections at the biceps brachii and triceps brachii (Fig. 1) using 180 paired pulses at 0.2 Hz, lasting 15 min. In the main experiment, MEPs were recorded before Hebbian stimulation (baseline) and after Hebbian stimulation at 0, 15, and 30 min postintervention (Post0, Post15, and Post30).

### TMS

Transcranial magnetic stimuli were delivered from a DuoMAG-MP Dual (DEYMED Diagnostic s.r.o., Hronov, Czech Republic) through a figure-of-eight coil (XT-70BF) with a biphasic current waveform. TMS was applied over the arm representation of the primary motor cortex, with the coil positioned at the optimal site for eliciting MEPs in the contralateral biceps and triceps brachii. To identify the optimal scalp position for each muscle, the coil was held tangential to the scalp, with the handle angled 45° away from the midline, and moved in small steps along the arm motor representation. Consistent with previous studies (4, 18, 19), the optimal scalp position for eliciting MEPs in both the biceps and triceps over-lapped. Maximum MEP amplitude (MEP-max) was measured by progressively increasing the intensity up to 100% of the maximum stimulator output (MSO). MEP-max referred to the largest MEP amplitude recorded in a single trial during incremental increases in stimulation intensity. Consistent with prior studies (20–23), our results indicate that MEP-max was reached at intensities below 100% MSO (Table 1). RMT was defined as the stimulator intensity needed to elicit MEPs with peak-to-peak amplitude ≥50 μV in 3 out of 5 consecutive trials at rest. No difference was seen between biceps and triceps in RMT values (biceps = 61.92 ± 15.71% of the MSO, triceps = 71.43 ± 17.74% of the MSO; *P* = 0.148, *d* = 0.6). Participants wore a cap on which the position of the coil was marked to ensure consistent stimulation location throughout the experiment. For cervical root (C-root) measurements, TMS was used to elicit MEPs through stimulation of the C5–C6 cervical spinal processes (7, 12).

### PNS

Transcutaneous electrical stimulation of the brachial plexus was applied using a DS7R stimulator (Digitimer Ltd, UK) with a 200 μs pulse width. The cathode was placed over the Erb’s point, and the anode was placed over the acromion. The maximal motor response (M-max) was determined by increasing the stimulus intensity until no increase in M-wave amplitude was observed. The M-max was measured as the peak-to-peak amplitude in mV of the nonrectified response. M-max measurements were taken for both biceps and triceps simultaneously until M-max was reached for both muscles. No difference was observed between M-max values in biceps (13.98 ± 6.30 mV) and triceps (9.93 ± 5.25 mV; *P* = 0.11). The stimuli delivered during intervention were supramaximal, set at 120% of the higher stimulus intensity between the two muscles.

### Hebbian Stimulation

**Figure 2.**
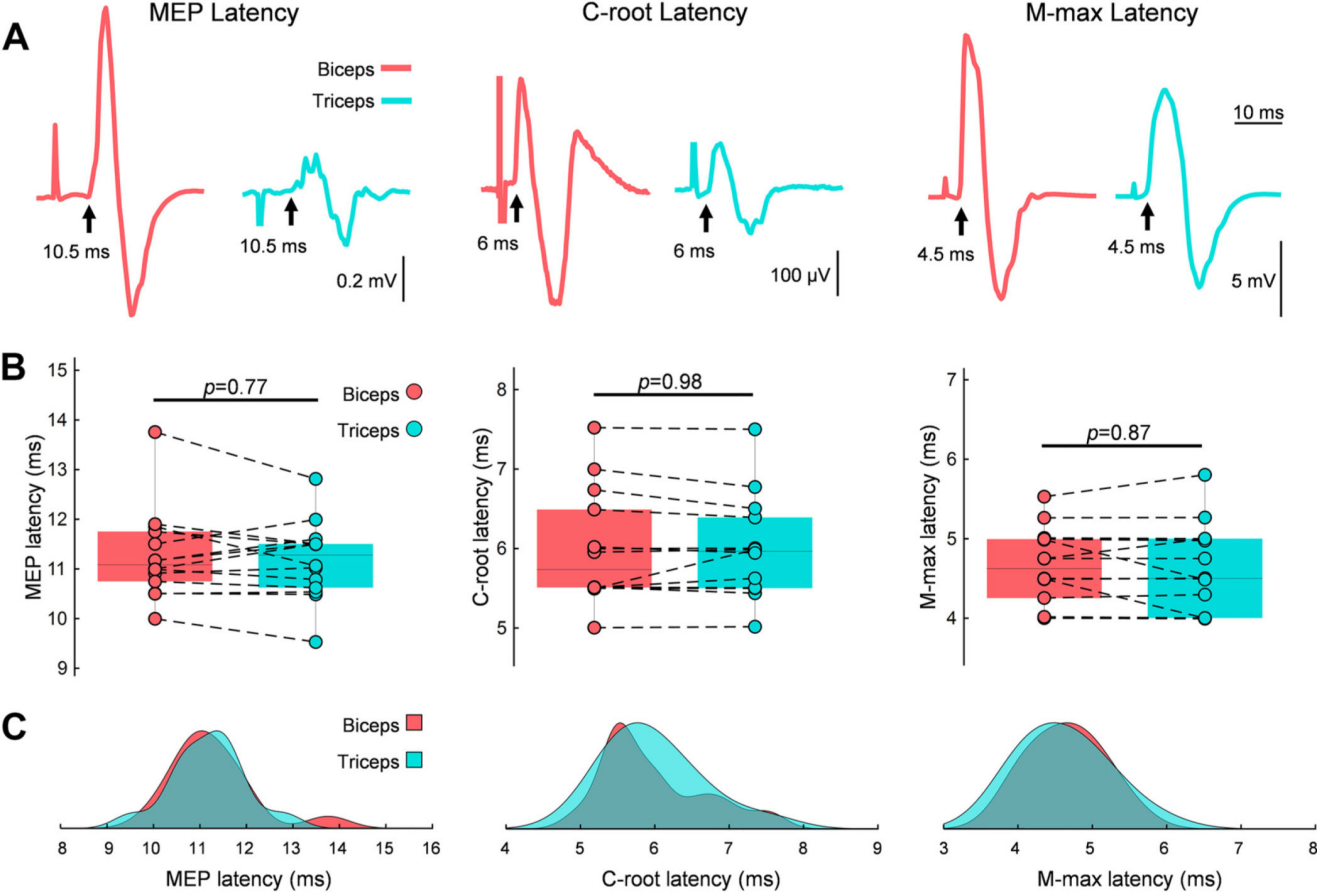
Latency measures in biceps and triceps brachii. *A*: central conduction time (CCT) and peripheral conduction time (PCT) were calculated using latencies from motor-evoked potentials (MEPs; *left*), C-root responses (*center*), and maximal motor responses (M-max; *right*). Representative traces from the biceps (pink) and triceps (blue) muscles are shown, with latencies indicated by arrows. *B*: box plot charts display group data. The *x*-axis represents the tested muscle (biceps in pink and triceps in blue), whereas the *y*-axis represents latency (ms). The *top* and *bottom* of each box correspond to the 75th percentile (*top quartile*) and 25th percentile (*bottom quartile*), respectively. The horizontal line within each box denotes the median (50th percentile). Vertical bars extend to the maximum and minimum values, and dotted black lines connect individual subject data (*n* = 14) between muscles. *C*: distribution plots illustrate the probability density of latency values (ms) for each measurement in the biceps (pink) and triceps (blue).

During Hebbian stimulation, performed while participants were at rest, the interstimulus interval (ISI) between TMS and PNS was set to target corticospinal-motoneuronal synapses with a 1–2 ms delay between pre- and postsynaptic activation. TMS intensity was set at 100% of the MSO, and the PNS intensity was set at 120% of the M-max. The ISI was determined by calculating the central conduction time (CCT), which represents the time for descending volleys elicited by TMS at the primary motor cortex to corticospinal neurons at the spinal level (referred to as presynaptic terminal), and peripheral conduction time (PCT), which represents the time for volleys elicited by the PNS at the brachial plexus to reach the motoneurons (postsynaptic terminal). CCT, PCT, and ISI were calculated for each subject according to the following equations: CCT = MEP latency - (C-root latency + 1.5); PCT = C-root latency - M-max latency + 0.5; ISI = CCT + 1.5 - PCT (Fig. 2). The estimated time for synaptic transmission and conduction to the nerve root at the vertebral foramina is ~1.5 ms (24, 25), and the estimated time for antidromic signal propagation from the vertebral foramina to the dendrites is ~0.5 ms (26). MEP latency was recorded during isometric contraction at 10% of the MVC using TMS with the intensity set to ~120% of the RMT. A waveform average of 20 responses was used to determine the latency, identified as the time when the response exceeded 2 standard deviations (SD) of the mean rectified background activity (measured 100 ms before the TMS stimulus artifact). C-root and M-max latencies were measured at rest using waveform averages of 10 and 5 responses, respectively. Onset latencies were verified by visual inspection to ensure a clear turning point. ISI was calculated for both biceps and triceps, and since no differences were found between muscles (Fig. 2), Hebbian stimulation was delivered with the triceps ISI.

### MEPs

At baseline, MEPs were assessed using two stimulation intensities: RMT (MEP-RMT) and 100% of the MSO (MEP-100%). These measurements were taken immediately and up to 30 min poststimulation in all participants (Table 1). Since the MEP-100% size was larger in the biceps than in the tri-ceps (biceps = 553.7 ± 453.4 μV, triceps = 160.4 ± 136.2 μV; *P* = 0.001, *d* = 0.92; Table 1), we conducted a control experiment (*n* = 11) testing responses of an intermediate size between MEP-RMT and MEP-100%. This intermediate size was specifically selected to fall on the upward slope of the recruitment curve for both the biceps and triceps muscles—a range that is particularly sensitive for detecting changes in corticospinal excitability (27)—while ensuring that the evoked amplitudes were similar in both muscles (referred to as MEP-Control; see Table 1). The same stimulus intensity values were used after Hebbian stimulation at Post0, Post15, and Post30 measurements. Twenty MEPs were recorded and averaged at rest for each measurement, and trials in which the background EMG activity was ≥20 μV were excluded from the analysis. The primary outcome measures included changes in MEP-RMT, MEP-Control, and MEP-100% from pre- to poststimulation, expressed as a percentage.

### Data Analysis

Normal distribution was assessed using Shapiro–Wilk’s test, whereas homogeneity of variances was tested using the Levene’s test of equality and Mauchly’s test of sphericity. When sphericity could not be assumed, the Greenhouse–Geisser correction statistic was used. If data were not normally distributed, a log transformation was performed, and normality was reassessed. If normality was confirmed after the transformation, parametric analysis was conducted; otherwise, nonparametric analysis (Mann–Whitney *U* test) was used. To account for repeated measures within each participant, a linear mixed effects model was fitted with the participant as the random intercept and amplitudes normalized to the baseline. First, we used fixed factors of TIME (Baseline, Post0, Post15, and Post30) and SIZE (MEP-RMT, MEP-100%) to assess biceps and triceps measurements. Second, we compared both muscles using MEP-Control and MEP-RMT amplitudes using fixed factors of TIME and MUSCLE (biceps and triceps). If no significant interaction was detected, the model was simplified by removing the interaction term and interpreting main effects. Bonferroni correction was used for all post hoc comparisons. Pearson correlation analysis was used as needed. A Student’s *t* test was used to compare values of MEP, C-root, and M-max latencies; MVC, M-max, and MEP-max; and unnormalized baseline MEP-RMT, MEP-Control, and MEP-100% amplitudes between the biceps and triceps. Statistical analyses were performed using SPSS (IBM SPSS Statistics for Windows, v. 26.0. Armonk, NY: IBM Corp). Group data are presented as the means ± SD, and *P* < 0.05 was considered statistically significant.

## RESULTS

### MEPs in the Biceps Brachii

**Figure 3.**
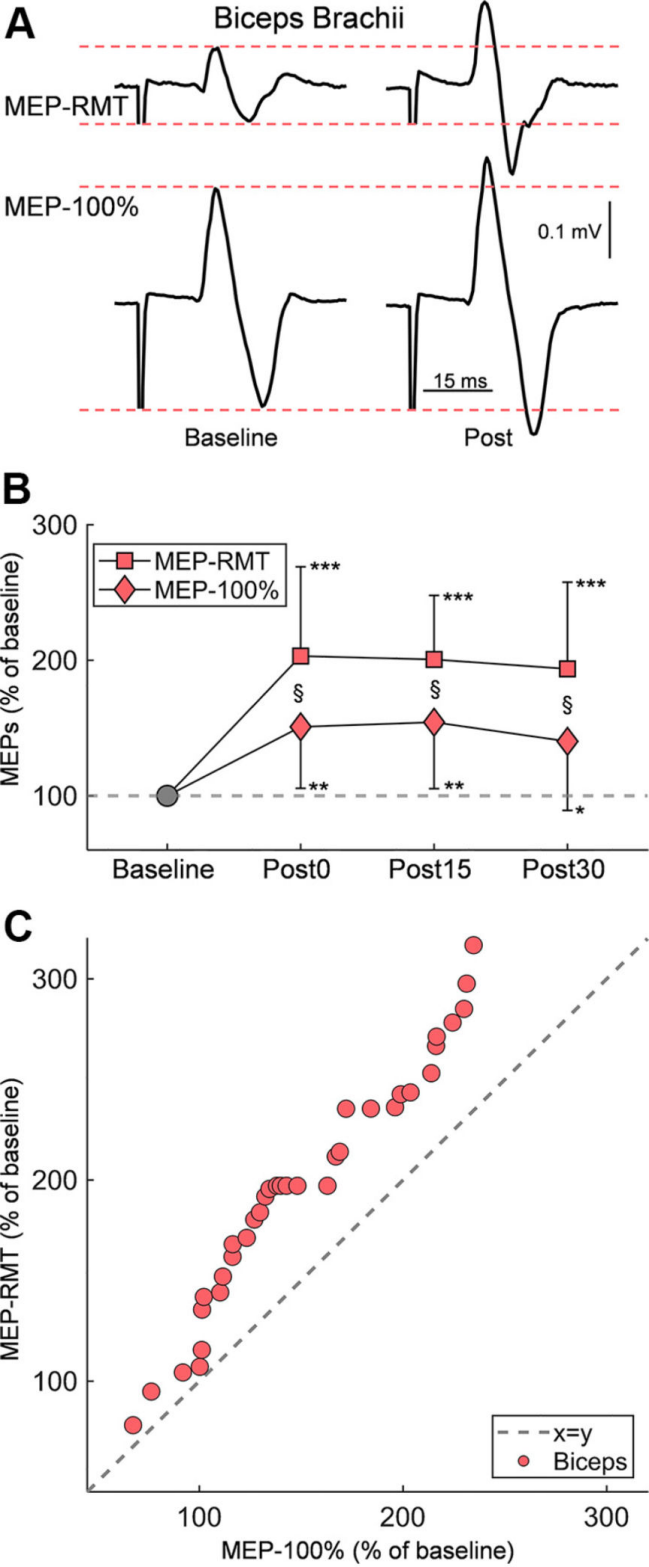
MEP change in biceps brachii. *A*: representative EMG traces of MEPs elicited by TMS in the biceps brachii are shown at baseline (*left*) and postintervention (*right*). Traces are displayed for two stimulus intensities: resting motor threshold (MEP-RMT; *top*) and maximum intensity (MEP-100%; *bottom*). *B*: the line plot presents group data, with MEP size expressed as a percentage of baseline amplitude (*y*-axis) across different time points (*x*-axis): baseline (gray marker) and postintervention at three intervals (Post0, Post15, and Post30). Mean values are indicated for MEP-RMT (square markers) and MEP-100% (diamond markers), with error bars representing standard deviation. *C*: the correlation plot displays individual data (*n* = 14) for each postintervention measurement (pink dots) assessed in the biceps. The dotted gray line (*x* = *y*) represents equality between the two measurements. Significance markers: *P < 0.05, **P < 0.01, ***P < 0.001 for comparisons with baseline; § for size comparisons between MEP-RMT and MEP-100%. EMG, electromyogram; MEP, motor-evoked potential; RMT, resting motor threshold; TMS, transcranial magnetic stimulation.

Figure 3A presents raw traces of MEP-RMT and MEP-100% recorded from the biceps brachii in a representative participant. Notably, both MEP-RMT and MEP-100% showed an increase in amplitude following Hebbian stimulation, as illustrated by traces recorded at 30 min poststimulation (Post30). A linear mixed model showed an effect of SIZE (*F*_1,85_ = 10.82, *P* = 0.0015) and TIME (*F*_3,85_ = 15.69, *P* < 0.001), but not in their interaction (*F*_3,85_ = 1.410, *P* = 0.245), on MEP amplitude in the biceps. Post hoc tests showed the increase in MEP-RMT (199.1 ± 58.2%) was significantly greater than the increase in MEP-100% (148.3 ± 47.7%; *P* = 0.0015) when data from all time points were grouped together. Notably, 12 out of 14 participants exhibited an increase in MEP-RMT amplitude at each assessed time point, and 11 out of 14 participants exhibited an increase in MEP-100% amplitude at each assessed time point. We found that the MEP-RMT amplitude increased significantly at Post0 (203.1 ± 66.0%, *P* < 0.001), Post15 (200.6 ± 47.3%, *P* < 0.001), and Post30 (193.7 ± 64.0%, *P* < 0.001) compared with baseline, and the MEP-100% amplitude increased significantly at Post0 (150.9 ± 45.4%, *P* = 0.0016), Post15 (154.3 ± 49.0%, *P* = 0.0018), and Post30 (140.2 ± 51.0%, *P* = 0.011) compared with baseline. Note that MEP-RMT amplitude increased significantly in MEP-RMT compared with MEP-100% at Post0 (*P* = 0.028), Post15 (*P* = 0.022), and Post30 (*P* = 0.025).

### MEPs in the Triceps Brachii

**Figure 4.**
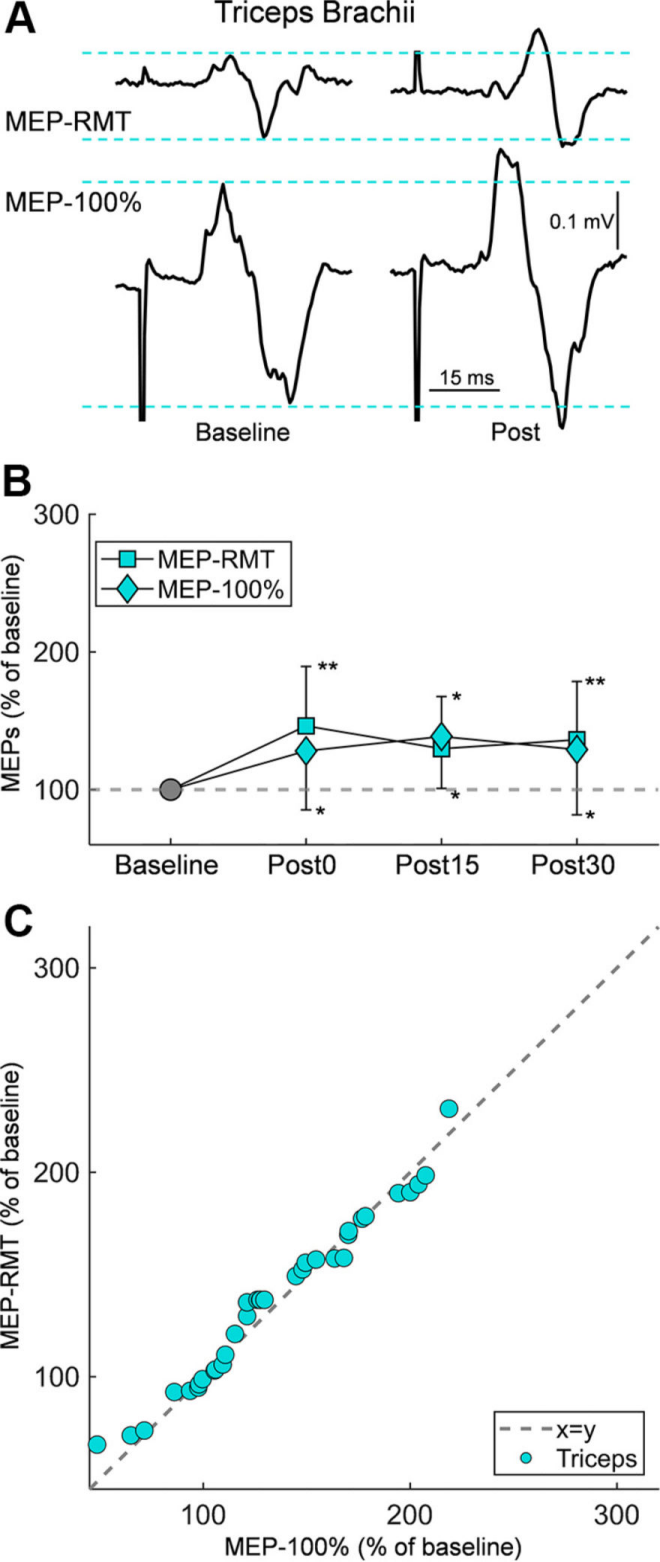
MEP change in triceps brachii. *A*: representative EMG traces of MEPs elicited by TMS in the triceps brachii are shown at baseline (*left*) and postintervention (*right*). Traces are displayed for two stimulus intensities: resting motor threshold (MEP-RMT; *top*) and maximum intensity (MEP-100%; *bottom*). *B*: the line plot presents group data, with MEP size expressed as a percentage of baseline amplitude (*y*-axis) across different time points (*x*-axis): baseline (gray marker) and postintervention at three intervals (Post0, Post15, and Post30). Mean values are indicated for MEP-RMT (square markers) and MEP-100% (diamond markers), with error bars representing standard deviation. *C*: the correlation plot dis-plays individual data (*n* = 14) for each postintervention measurement (blue dots) assessed in the triceps. The dotted gray line (*x* = *y*) represents equality between the two measurements. Significance markers: *P < 0.05, **P < 0.01 for comparisons with baseline. EMG, electromyogram; MEP, motor-evoked potential; RMT, resting motor threshold; TMS, transcranial magnetic stimulation.

Figure 4A presents raw traces of MEP-RMT and MEP-100% recorded from the triceps brachii in a representative participant. Both MEP-RMT and MEP-100% showed a similar increase in amplitude following Hebbian stimulation, as illustrated by traces recorded at 30-min poststimulation (Post30). A linear mixed model showed no effect of SIZE (*F*_1,85_ = 0.126, *P* = 0.724), but an effect of TIME (*F*_3,85_ = 5.846, *P* = 0.0011), and no effect in their interaction (*F*_3,85_ = 0.898, *P* = 0.446) on MEP amplitude in the triceps. Post hoc tests showed the increase in MEP-RMT (137.2 ± 40.6%) was not different than the increase in MEP-100% (131.7 ± 42.2%; *P* = 0.724; Fig. 4B). Notably, 10 out of 14 participants exhibited an increase in MEP-RMT amplitude at each assessed time point, and 7 out of 14 participants exhibited an increase in MEP-100% amplitude at each assessed time point. We found that the MEP-RMT amplitude increased significantly at Post0 (146.3 ± 43.2%, *P* = 0.0023), Post15 (129.8 ± 37.9%, *P* = 0.011), and Post30 (136.2 ± 42.3%, *P* = 0.0094) compared with base-line, and the MEP-100% amplitude increased significantly at Post0 (128.1 ± 42.8%, *P* = 0.035), Post15 (138.6 ± 37.7%, *P* = 0.0046), and Post30 (129.1 ± 47.4%, *P* = 0.039) compared with baseline. In addition, MEP-RMT amplitude was not different compared with MEP-100% at Post0 (*P* = 0.292), Post15 (*P* = 0.558), and Post30 (*P* = 0.684).

### MEPs in the Biceps versus Triceps Brachii

Because the maximum MEP size was larger in the biceps than in the triceps, we conducted a control experiment testing responses at an intermediate size referred to as MEP-Control, ensuring similar baseline sizes between muscles. This subgroup of participants (*n* = 11) showed similar baseline amplitudes at both MEP-RMT (*P* = 0.255) and MEP-Control (*P* = 0.230) for the control experiment.

**Figure 5.**
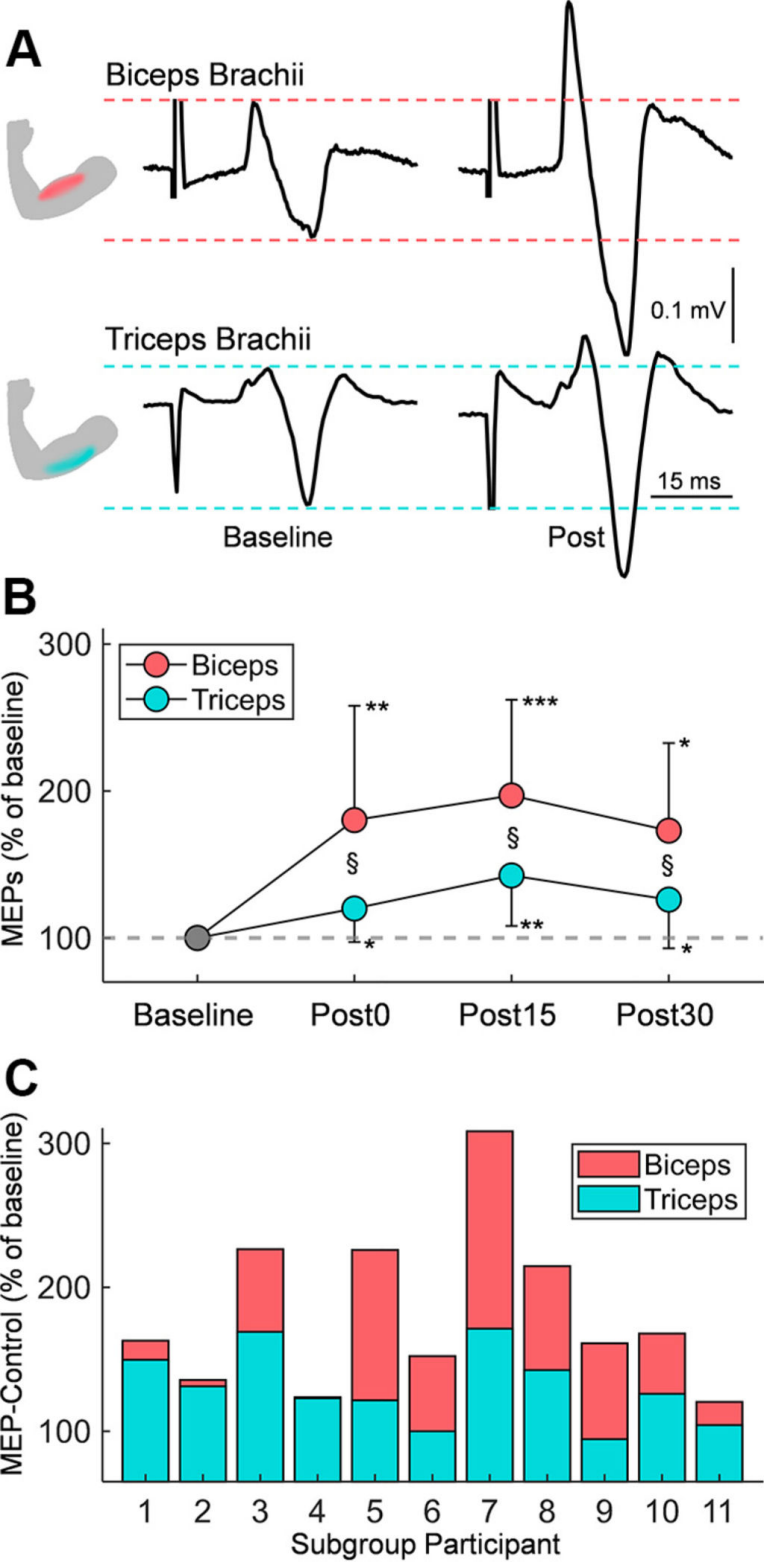
MEP changes between muscles. *A*: representative EMG traces of MEPs in the biceps brachii (*top*) and triceps brachii (*bottom*) in a participant, showing MEPs elicited by TMS at baseline (*left*) and postintervention (*right*). *B*: line plots display the increase in MEP amplitude in group data comparing muscles with matched baseline sizes. The *y*-axis represents MEP size as a percentage of baseline amplitude, and the *x*-axis shows the timeline of measurements: before intervention (Base, 100%) and after intervention at three time points (Post0, Post15, and Post30). Mean values are shown as circles, with the biceps in pink and the triceps in blue. Error bars indicate the standard deviation. *C*: bar plot illustrates the mean increase in MEP-Control amplitude (% of baseline) for each participant (*x*-axis; *n* = 14) in the biceps (pink) and triceps (blue). Significance markers: *P < 0.05, **P < 0.01, ***P < 0.001 for comparisons with baseline; § for muscle comparison (biceps vs. triceps). EMG, electromyogram; MEPs, motor-evoked potentials; TMS, transcranial magnetic stimulation.

Figure 5A presents raw traces of MEP-Control recorded from the biceps and triceps brachii in a representative participant. Notably, MEP-Control increases in amplitude following Hebbian stimulation, as illustrated by traces recorded at 30 min poststimulation (Post30). A linear mixed model showed an effect of TIME (*F*_3,76_ = 9.172, *P* < 0.001) and MUSCLE (*F*_1,76_ = 10.37, *P* = 0.0019), but not in their interaction (*F*_3,76_ = 1.581, *P* = 0.200), on MEP-Control amplitude. Post hoc analysis showed a larger MEP increase in biceps (180.8 ± 53.8%) than triceps (134.2 ± 34.7%; *P* = 0.0019) when data from all time points were grouped together. We found that the biceps MEP amplitude increased significantly at Post0 (180.2 ± 78.0%, *P* = 0.0098), Post15 (197.0 ± 65.2%, *P* < 0.001), and Post30 (173.1 ± 59.6%, *P* = 0.0037) compared with baseline, and triceps MEP amplitude increased significantly at Post0 (120.0 ± 22.8%, *P* = 0.015), Post15 (142.5 ± 34.4%, *P* = 0.0021), and Post30 (126.1 ± 33.1%, *P* = 0.034) compared with baseline. We observed that 10 out of 11 participants exhibited a larger increase in MEP-Control amplitude in the biceps than the triceps (Fig. 5C). Post hoc analysis showed larger MEP-Control in biceps than triceps at Post0 (*P* = 0.039), Post15 (*P* = 0.027), and Post30 (*P* = 0.047).

In addition, we compared MEP-RMT between muscles in the group that completed the experiment subgroup (*n* = 11). A linear mixed model showed an effect of TIME (*F*_3,76_ = 12.61, *P* < 0.001) and MUSCLE (*F*_1,76_ = 17.95, *P* < 0.001), but not in their interaction (*F*_3,76_ = 2.444, *P* = 0.071), on MEP-RMT amplitude. Post hoc analysis showed a larger MEP-RMT increase in biceps (171.3 ± 69.5%) than triceps (129.1 ± 42.1%; *P* = 0.0025) when data from all time points were grouped together. We observed that 10 out of 11 participants exhibited a larger increase in MEP-RMT amplitude in the biceps than in the triceps. Post hoc analysis showed larger MEP-RMT in biceps than triceps at Post0 (*P* = 0.017), Post15 (*P* = 0.016), and Post30 (*P* = 0.021).

## DISCUSSION

Our findings provide evidence of muscle-specific effects of Hebbian stimulation in neurologically intact humans. We observed that both MEP-RMT and MEP-100% increased in the biceps and triceps immediately and up to 30 min poststimulation. However, the increase in MEP-RMT was significantly greater in the biceps compared with the triceps, whereas MEP-100% changes were comparable between muscles. To confirm this effect between muscles, we conducted a control experiment assessing responses at an intermediate size (MEP-Control) with matched baseline amplitudes between muscles. Notably, Hebbian stimulation continued to elicit a greater increase in MEP-Control in the biceps than in the triceps. These findings indicate that Hebbian stimulation preferentially enhances corticospinal excitability in elbow flexors over extensors. This muscle-specific response highlights the importance of considering muscle-specific innervation pat-terns when future studies assess the therapeutic effect of this technique in individuals with motor impairment.

### Hebbian Stimulation in the Biceps and Triceps Brachii

Consistent with previous studies (28, 29), we found that Hebbian stimulation effectively enhanced corticospinal excitability in the biceps brachii. For the first time, we investigated the effects of Hebbian stimulation in the triceps brachii and directly compared its impact between two muscles. Although MEP size increased in the triceps following stimulation, the enhancement was significantly greater in the biceps, highlighting a differential effect of Hebbian stimulation between elbow flexors and extensors. To better evaluate muscle-specific differences, we compared the effects of Hebbian stimulation on MEPs of two distinct sizes. One was near the threshold (MEP-RMT), where low-intensity TMS predominantly recruits low-threshold motoneurons. Evidence suggests that TMS recruits motoneurons in accordance with the Henneman size principle, whereby smaller motor units are activated first, followed by larger ones as force production increases, regardless of the source of excitation (30, 31). We observed greater increases in MEP-RMT in the biceps compared with the triceps brachii, suggesting a muscle-specific effect of Hebbian stimulation on smaller motoneurons. Given that Hebbian stimulation likely targets small motoneurons activated during low levels of voluntary contraction (12, 29), it is possible that MEP-RMT is more sensitive to this form of plasticity due to its predominant composition of small motoneurons. Since MEP-100% changes did not differ between muscles, the smaller effects observed in this outcome compared with MEP-RMT may be due to the combined contribution of both small and large motoneurons to MEP-100%. It is likely that the size of MEP-RMT predominantly recruited low-threshold motoneurons compared with MEP-100%, making them more sensitive to Hebbian stimulation as previously suggested (12, 29).

A critical question is why MEP-RMT and MEP-Control, both of which reflect MEP sizes less likely to be saturated, detected larger increases in corticospinal excitability in the biceps but not in the triceps brachii. Evidence has shown that descending input from corticospinal neurons provides more facilitatory monosynaptic connections to the biceps, whereas the triceps receive greater disynaptic inhibitory input (3, 32). PSTHs have demonstrated that monosynaptic corticospinal facilitation is more pronounced in elbow flexor motoneurons than in elbow extensor motoneurons (3). Thus, another possibility is that we have more chances to elicit this type of plasticity in the biceps due to a larger number of cortico-spinal-motoneuronal connections. Another possibility is that differences between flexor and extensor motoneurons contributed to our findings. In humans, H-reflexes (reflecting motoneuron excitability) can be elicited in the biceps brachii (33) but can be difficult or impossible to elicit in the triceps brachii (34). However, persistent inward currents that also reflect motoneuron excitability (a prolonged excitation of motoneurons without requiring additional descending input) are more pronounced in extensor motoneurons compared with flexor motoneurons in animals (35, 36) and humans (37), making it less clear that differences in excitability between motoneuron pools contributed to our findings.

Our results agree with findings showing that plasticity induced by pairing auditory and muscle stimulation using principles of STDP increases in corticospinal excitability predominantly in elbow flexor muscles compared with elbow extensor muscles (14, 15). Also, repetitive practice of a tracking task involving finger flexor and extensor muscles resulted in increases in corticospinal excitability in flexor muscles but not in extensor muscles (17). In addition, when plasticity is induced by sensory input, pairing motor point stimulation of a finger flexor or extensor muscle with TMS of the motor cortex enhances corticospinal excitability more in flexor muscles than in extensor muscles (16). When sensory input was inhibited using an ischemic nerve block, MEP amplitude increased in wrist flexors but not in wrist extensors (38), suggesting a differential influence of afferent input on corticospinal excitability between muscle groups. Indeed, differences have been reported in the role of afferent input onto elbow flexor and extensor motoneurons, both from homonymous and heteronymous muscles (39), as well as from afferents acting across different joints (40). Notably, afferent input has been shown to facilitate biceps motoneurons while depressing triceps motoneurons (40). Although PNS during Hebbian stimulation likely activated afferent fibers and motoneurons of both muscles to a similar extent—since afferent input was maintained constant with the same stimulus intensity targeting both muscles concurrently—because the stimulus intensity was supramaximal, we cannot entirely rule out the contribution from afferent input from nearby joints to our findings. However, if afferent input were a major factor, we would have expected a greater increase in MEP-100% for the biceps than for the triceps, which was not observed. This interpretation aligns with previous findings indicating that the magnitude of reciprocal Ia inhibition—assessed by activating group Ia afferent fibers from the antagonistic muscle—is similar between elbow flexor and extensor motoneurons (41). Therefore, regardless of the specific mechanisms underlying our results, our findings support the possibility that low-threshold motoneurons contributed to these effects.

### Functional Considerations

Several studies have demonstrated the potential for Hebbian stimulation to not only temporarily increase corticospinal excitability in targeted muscles in both neurologically intact individuals and those with spinal cord injury (12, 13, 42, 43) but also to enhance motor recovery (7–9). Our study suggests that muscle-specific innervation patterns may be a relevant factor to consider when developing therapeutic strategies aimed at improving upper limb function in individuals with motor impairment. At present, most neurostimulation protocols apply similar stimulation doses across different muscle groups. Exploring whether dosage effects differ between muscles could be a valuable direction for future research involving individuals with spinal cord injury and other motor impairments. This study also highlights the challenges of inducing plasticity in the triceps brachii. Similar difficulties have been observed in patients with spinal cord injury, where motor recovery in elbow extensors is more impaired than in flexors. This disparity may be partly attributed to corticospinal pathway weakness (5). Flexor motoneurons might also be more sensitive to use-dependent plasticity. For example, flexor muscles demonstrate superior control in precise movements compared with extensors (44) and individuation tasks (45). Likewise, in tennis players, forehand strokes (arm flex-ion) exhibit greater accuracy than backhand strokes [arm extension (46)]. Given that deficits in upper limb extensors are common in various neurological conditions (i.e., stroke and spinal cord injury), identifying strategies to optimize or supplement Hebbian stimulation for more effective engagement of the triceps is a critical area for future research.

### Limitations of the Study

When interpreting our results, it is important to consider some potential limitations, including differences in MEP response size between the biceps and triceps. However, it is unlikely that stimulus intensity during Hebbian stimulation played a role in our findings, as both muscles exhibited similar RMT values. Another possibility is that the size of MEPs at baseline affected our findings. Indeed, the size of the maximum MEP was larger in the biceps than in the triceps, consistent with previous results (4, 5). To address this point, we conducted a control experiment in which we tested MEP responses at an intermediate size between MEP-RMT and MEP-100% (MEP-Control), ensuring similar baseline sizes between muscles that were in the ascending part of the input-output recruitment excitability curve. In agreement with our results during MEP-RMT comparisons, we observed that Hebbian stimulation continued to produce a greater increase in MEP-Control in the biceps than in the triceps. It is also important to note that, although our power analysis indicated that 14 participants were sufficient to test our hypothesis, the sample size remains relatively small, and the results should therefore be interpreted with caution.

## Conclusions

Our results suggest that Hebbian plasticity enhances corticospinal excitability to a greater extent in elbow flexor than extensor muscles, highlighting the importance of considering muscle-specific innervation patterns in future studies evaluating the therapeutic potential of this technique in individuals with motor impairment, such as spinal cord injury and stroke.

## Figures and Tables

**Table 1.** MEP baseline amplitudes

	Biceps, μV	Triceps, μV	*P* Value

MEP-RMT	56.25 ± 20.51	42.28 ± 19.69	0.103
MEP-Control	211.8 ± 170.0	127.2 ± 109.6	0.230
MEP-100%	553.7 ± 453.4	160.4 ± 136.2	**0.007**
MEP-max	900.7 ± 686.6	283.1 ± 214.6	**0.010**

Boldface *P* values represent significance of *P* < 0.05. MEP, motor-evoked potential; RMT, resting motor threshold.

## Data Availability

Data will be made available upon reasonable request.
